# Increased Methamphetamine, Injection Drug, and Heroin Use Among Women and Heterosexual Men with Primary and Secondary Syphilis — United States, 2013–2017

**DOI:** 10.15585/mmwr.mm6806a4

**Published:** 2019-02-15

**Authors:** Sarah E. Kidd, Jeremy A. Grey, Elizabeth A. Torrone, Hillard S. Weinstock

**Affiliations:** 1 Division of STD Prevention, National Center for HIV/AIDS, Viral Hepatitis, STD, and TB Prevention, CDC.

During 2013–2017, the national annual rate of reported primary and secondary (P&S) syphilis cases in the United States increased 72.7%, from 5.5 to 9.5 cases per 100,000 population (*1*). The highest rates of P&S syphilis are seen among gay, bisexual, and other men who have sex with men (collectively referred to as MSM) (*2*), and MSM continued to account for the majority of cases in 2017 (*1*). However, during 2013–2017, the P&S syphilis rate among women increased 155.6% (from 0.9 to 2.3 cases per 100,000 women), and the rate among all men increased 65.7% (from 10.2 to 16.9 cases per 100,000 men), indicating increasing transmission between men and women in addition to increasing transmission between men (*1*). To further understand these trends, CDC analyzed national P&S syphilis surveillance data for 2013–2017 and assessed the percentage of cases among women, men who have sex with women only (MSW), and MSM who reported drug-related risk behaviors during the past 12 months. Among women and MSW with P&S syphilis, reported use of methamphetamine, injection drugs, and heroin more than doubled during 2013–2017. In 2017, 16.6% of women with P&S syphilis used methamphetamine, 10.5% used injection drugs, and 5.8% used heroin during the preceding 12 months. Similar trends were seen among MSW, but not among MSM. These findings indicate that a substantial percentage of heterosexual syphilis transmission is occurring among persons who use these drugs, particularly methamphetamine. Collaboration between sexually transmitted disease (STD) control programs and partners that provide substance use disorder services will be important to address recent increases in heterosexual syphilis.

P&S syphilis case report data were extracted from the National Notifiable Diseases Surveillance System, the system through which CDC receives syphilis and other notifiable sexually transmitted disease data from all 50 states and the District of Columbia. P&S syphilis case report data include demographic information and also risk factor information, such as information about sex partners and drug use within the past 12 months, which is obtained through case interviews or investigation by the local health department.

For this analysis, men with syphilis were categorized as MSM if they reported having sex with any male partner in the last 12 months; men who reported having sex with only female partners in the last 12 months were categorized as MSW. To assess drug-related behaviors, the following are included in the case report data as separate yes/no variables: use of injection drugs, methamphetamines, heroin, cocaine, crack, nitrates/poppers, erectile dysfunction drugs, other drugs, no drugs; and sex with a person who injects drugs within the last 12 months. The percentage of persons reporting use of each drug or behavior was calculated separately, using those with a “yes” response to the relevant variable as the numerator. For the injection drug use and sex with a person who injects drugs variables, the percentage of persons reporting these behaviors was calculated among persons with “yes” or “no” responses for that behavior (i.e., those with missing or unknown responses were excluded from the denominator). Because some local health departments collected data on the remaining drug use variables (e.g., methamphetamine and heroin use) differently and did not routinely report “no” responses to these variables, persons with missing and unknown responses for these remaining drug use variables were included in the denominator if they had a “yes” response to any of these variables and also did not have a “no” response to any of these variables (i.e., for these persons, missing and unknown responses were assumed to be “no” responses). SAS statistical software (version 9.3, SAS Institute Inc.) was used for all analyses.

During 2013–2017, the percentage of persons with P&S syphilis who reported methamphetamine use, sex with a person who injects drugs, injection drug use, or heroin use within the past 12 months more than doubled among women and MSW (Table 1). The percentage of persons with P&S syphilis reporting methamphetamine use increased from 6.2% to 16.6% among women, and from 5.0% to 13.3% among MSW, but decreased from 9.2% to 8.0% among MSM. The percentage of persons with P&S syphilis reporting sex with a person who injects drugs increased from 5.5% to 12.4% among women and from 3.6% to 9.3% among MSW, but increased only slightly among MSM (from 4.3% to 5.2%). Injection drug use increased from 4.0% to 10.5% among women with P&S syphilis and from 2.8% to 6.3% among MSW, but remained stable at 3.5% among MSM. Heroin use increased from 2.1% to 5.8% among women with P&S syphilis and from 0.8% to 2.7% among MSW, but remained relatively stable (increased from 0.7% to 0.8%) among MSM.

**TABLE 1:** Prevalence* of selected drug-related behaviors among women, men who have sex with women only (MSW), and men who have sex with men (MSM) with reported primary or secondary syphilis — National Notifiable Diseases Surveillance System, United States, 2013–2017

Behavior during past 12 months	No. (%)				
	2013	2014	2015	2016	2017
**Used methamphetamine**					
Women	69 (6.2)	92 (6.8)	184 (10.6)	317 (13.7)	456 (16.6)
MSW	88 (5.0)	151 (7.4)	194 (7.6)	347 (11.1)	482 (13.3)
MSM	805 (9.2)	867 (8.7)	855 (7.5)	1,039 (7.9)	1,132 (8.0)
**Total^†^**	**987 (7.9)**	**1,136 (7.9)**	**1,253 (7.4)**	**1,738 (8.5)**	**2,106 (9.6)**
**Had sex with person who injects drugs**					
Women	64 (5.5)	113 (8.3)	135 (7.9)	217 (9.9)	325 (12.4)
MSW	64 (3.6)	119 (5.8)	167 (6.4)	201 (6.6)	325 (9.3)
MSM	368 (4.3)	495 (5.0)	537 (4.7)	594 (4.6)	725 (5.2)
**Total^†^**	**499 (4.2)**	**734 (5.3)**	**847 (5.2)**	**1,015 (5.5)**	**1,380 (6.7)**
**Used injection drugs**					
Women	44 (4.0)	81 (6.1)	119 (7.0)	179 (8.1)	281 (10.5)
MSW	48 (2.8)	78 (3.7)	96 (3.6)	152 (4.8)	230 (6.3)
MSM	288 (3.5)	365 (3.6)	345 (2.9)	406 (3.1)	514 (3.5)
**Total^†^**	**388 (3.5)**	**534 (3.8)**	**569 (3.4)**	**745 (3.9)**	**1,042 (4.9)**
**Used heroin**					
Women	23 (2.1)	42 (3.1)	59 (3.4)	109 (4.7)	156 (5.8)
MSW	15 (0.8)	37 (1.8)	44 (1.7)	66 (2.1)	97 (2.7)
MSM	57 (0.7)	49 (0.5)	78 (0.7)	102 (0.8)	117 (0.8)
**Total^†^**	**95 (0.8)**	**131 (0.9)**	**182 (1.1)**	**279 (1.4)**	**375 (1.7)**

* Calculated among persons for whom data for that behavior were reported (persons with missing or unknown responses were excluded from the denominator).

^†^ Includes case records with unknown sex and men with unknown data on sex of sex partner.

Among women with P&S syphilis, increases in methamphetamine use, sex with a person who injects drugs, injection drug use, and heroin use were observed in every region of the United States (Table 2). Among MSW with P&S syphilis, the increase in sex with a person who injects drugs was observed in every region, and the increases in methamphetamine, injection drug, and heroin use occurred in all regions except the Northeast (Table 3). Although trends were generally similar across regions, the prevalence of these behaviors among women and MSW with P&S syphilis varied considerably by region. In 2017, the percentages of both women and MSW reporting these behaviors were highest in the West and lowest in the Northeast. In the West, methamphetamine use during the past 12 months was reported by 34.8% of women with P&S syphilis and 25.0% of MSW with P&S syphilis. In addition, 22.6% of women with P&S syphilis in the West had sex with a person who injects drugs, and 21.2% used injection drugs (Table 2). In contrast, <3% of women or MSW with P&S syphilis in the Northeast reported these behaviors in 2017 (Table 2) (Table 3). Additional data on other behaviors and characteristics reported among persons with P&S syphilis, such as number of sex partners, HIV status, and other drug use data, are available online in a supplemental syphilis surveillance report (https://www.cdc.gov/std/stats17/syphilis2017/).

**TABLE 2:** Prevalence* of selected drug-related behaviors among women with reported primary and secondary syphilis, by U.S. Census region^†^ — National Notifiable Diseases Surveillance System, United States, 2013–2017

Behavior during past 12 months/Region	No. (%)				
	2013	2014	2015	2016	2017
**Used methamphetamine**					
West	50 (21.7)	63 (19.2)	119 (26.8)	230 (30.7)	310 (34.8)
Midwest	1 (0.8)	6 (3.4)	11 (6.6)	18 (7.7)	31 (13.0)
South	18 (2.7)	22 (3.0)	54 (5.5)	68 (6.0)	112 (8.0)
Northeast	0 (0.0)	1 (0.8)	0 (0.0)	1 (0.5)	3 (1.4)
**Total women**	**69 (6.2)**	**92 (6.8)**	**184 (10.6)**	**317 (13.7)**	**456 (16.6)**
**Had sex with person who injects drugs**					
West	28 (14.5)	50 (18.4)	56 (16.6)	104 (20.8)	140 (22.6)
Midwest	10 (4.9)	17 (6.8)	13 (4.7)	37 (11.7)	57 (16.4)
South	26 (3.8)	39 (5.4)	62 (6.5)	71 (6.0)	122 (8.6)
Northeast	0 (0.0)	7 (6.0)	4 (2.8)	5 (2.6)	6 (2.7)
**Total women**	**64 (5.5)**	**113 (8.3)**	**135 (7.9)**	**217 (9.9)**	**325 (12.4)**
**Used injection drugs**					
West	19 (17.3)	36 (19.5)	47 (17.0)	73 (14.5)	134 (21.2)
Midwest	9 (4.5)	12 (5.0)	17 (6.5)	33 (10.6)	43 (12.5)
South	16 (2.2)	32 (4.1)	53 (5.2)	67 (5.6)	98 (6.7)
Northeast	0 (0.0)	1 (0.8)	2 (1.4)	6 (3.0)	6 (2.6)
**Total women**	**44 (4.0)**	**81 (6.1)**	**119 (7.0)**	**179 (8.1)**	**281 (10.5)**
**Used heroin**					
West	8 (3.5)	15 (4.6)	15 (3.4)	40 (5.4)	67 (7.8)
Midwest	1 (0.8)	8 (4.5)	7 (4.2)	14 (6.0)	9 (3.8)
South	14 (2.1)	18 (2.4)	36 (3.7)	49 (4.3)	75 (5.4)
Northeast	0 (0.0)	1 (0.8)	1 (0.7)	6 (3.1)	5 (2.3)
**Total women**	**23 (2.1)**	**42 (3.1)**	**59 (3.4)**	**109 (4.7)**	**156 (5.8)**

* Calculated among persons for whom data for that behavior were reported (persons with missing or unknown responses were excluded from the denominator).

^†^ *West*: Alaska, Arizona, California, Colorado, Hawaii, Idaho, Montana, Nevada, New Mexico, Oregon, Utah, Washington, and Wyoming; *Midwest*: Illinois, Indiana, Iowa, Kansas, Michigan, Minnesota, Missouri, Nebraska, North Dakota, Ohio, South Dakota, and Wisconsin; *South*: Alabama, Arkansas, Delaware, District of Columbia, Florida, Georgia, Kentucky, Louisiana, Maryland, Mississippi, North Carolina, Oklahoma, South Carolina, Tennessee, Texas, Virginia, and West Virginia; *Northeast*: Connecticut, Maine, Massachusetts, New Hampshire, New Jersey, New York, Pennsylvania, Rhode Island, and Vermont.

**TABLE 3:** Prevalence* of selected drug-related behaviors among men who have sex with women only (MSW) with reported primary and secondary syphilis, by U.S. Census region^†^ — National Notifiable Diseases Surveillance System, United States, 2013–2017

Behavior during past 12 months/Region	No. (%)				
	2013	2014	2015	2016	2017
**Used methamphetamine**					
West	55 (13.4)	112 (19.5)	121 (18.1)	232 (24.8)	313 (25.0)
Midwest	2 (1.1)	6 (2.4)	20 (8.1)	27 (8.5)	36 (11.6)
South	29 (2.9)	31 (3.0)	52 (3.8)	86 (5.6)	130 (7.6)
Northeast	2 (1.1)	2 (1.0)	1 (0.4)	2 (0.6)	3 (0.8)
**Total MSW**	**88 (5.0)**	**151 (7.4)**	**194 (7.6)**	**347 (11.1)**	**482 (13.3)**
**Had sex with person who injects drugs**					
West	19 (6.1)	56 (12.5)	61 (11.8)	80 (13.0)	126 (14.9)
Midwest	8 (2.5)	14 (3.5)	29 (6.2)	29 (6.0)	55 (11.5)
South	34 (3.4)	44 (4.5)	71 (5.2)	84 (5.3)	133 (7.6)
Northeast	3 (1.7)	5 (2.2)	6 (2.2)	8 (2.1)	11 (2.6)
**Total MSW**	**64 (3.6)**	**119 (5.8)**	**167 (6.4)**	**201 (6.6)**	**325 (9.3)**
**Used injection drugs**					
West	18 (10.2)	34 (8.5)	39 (8.8)	69 (10.2)	118 (13.0)
Midwest	3 (0.9)	6 (1.5)	13 (2.8)	24 (5.2)	22 (4.7)
South	24 (2.3)	35 (3.2)	39 (2.6)	51 (3.1)	85 (4.7)
Northeast	3 (1.6)	3 (1.3)	5 (1.7)	8 (2.0)	5 (1.1)
**Total MSW**	**48 (2.8)**	**78 (3.7)**	**96 (3.6)**	**152 (4.8)**	**230 (6.3)**
**Used heroin**					
West	4 (1.0)	12 (2.1)	16 (2.4)	26 (2.8)	48 (3.9)
Midwest	0 (0.0)	4 (1.6)	2 (0.8)	9 (2.8)	7 (2.3)
South	9 (0.9)	21 (2.1)	23 (1.7)	24 (1.6)	40 (2.4)
Northeast	2 (1.1)	0 (0.0)	3 (1.2)	7 (2.2)	2 (0.5)
**Total MSW**	**15 (0.8)**	**37 (1.8)**	**44 (1.7)**	**66 (2.1)**	**97 (2.7)**

* Calculated among persons for whom data for that behavior were reported (persons with missing or unknown responses were excluded from the denominator).

^†^ *West*: Alaska, Arizona, California, Colorado, Hawaii, Idaho, Montana, Nevada, New Mexico, Oregon, Utah, Washington, and Wyoming; *Midwest*: Illinois, Indiana, Iowa, Kansas, Michigan, Minnesota, Missouri, Nebraska, North Dakota, Ohio, South Dakota, and Wisconsin; *South*: Alabama, Arkansas, Delaware, District of Columbia, Florida, Georgia, Kentucky, Louisiana, Maryland, Mississippi, North Carolina, Oklahoma, South Carolina, Tennessee, Texas, Virginia, and West Virginia; *Northeast*: Connecticut, Maine, Massachusetts, New Hampshire, New Jersey, New York, Pennsylvania, Rhode Island, and Vermont.

## Discussion

Since reaching a historic low in the United States in 2000–2001, the annual national rate of reported P&S syphilis cases has increased, and the rate in 2017 (9.5 per 100,000 population) was the highest reported since 1993 (*1*). Until 2013, the increase was primarily among MSM, and rates of P&S syphilis among women remained low and relatively stable (*3*). However, during 2013–2017, the P&S syphilis rate increased among both men and women (*1*). This report demonstrates that, during this same period, the prevalences of methamphetamine use, sex with a person who injects drugs, injection drug use, and heroin use within the past 12 months more than doubled among MSW and women with P&S syphilis, but not among MSM with P&S syphilis.

These findings indicate that a substantial percentage of heterosexual syphilis transmission is occurring among persons who use methamphetamine, inject drugs or have sex with persons who inject drugs, or who use heroin, and that heterosexual syphilis and drug use are intersecting epidemics. A linkage between heterosexual syphilis and drug use has been observed previously. In the late 1980s and early 1990s, increases in heterosexual syphilis were associated with crack cocaine use (*4*,*5*). Drug use, particularly use of methamphetamine and injection drugs, is associated with sexual behaviors that increase the risk for acquiring syphilis and other sexually transmitted diseases, including having multiple sex partners or concurrent sexual partnerships, inconsistent condom use, and exchange of sex for drugs or money (*6–**8*). In addition, among persons who use drugs, stigma and mistrust of the health care system along with other social determinants of health (e.g., unstable housing, poverty, incarceration, and lack of health insurance or a medical home) might contribute to decreased health care utilization and reluctance or inability to identify and locate sex partners, resulting in delays in diagnosis and treatment (*4*,*5*). These complications likely contribute to increasing syphilis incidence in communities and pose significant challenges to syphilis prevention and control efforts.

Pilot projects have demonstrated the feasibility and benefit of implementing substance use disorder interventions in STD clinics (*9*,*10*). STD programs should consider partnering with substance use disorder prevention and treatment programs and other organizations that provide services to persons who use drugs in the local community. Heterosexual networks and sexual risk behaviors are linked with drug use, and STD programs should work with substance use programs to facilitate referrals to substance use disorder treatment services when needed and to integrate STD and substance use disorder prevention and treatment services when possible. Substance use disorder programs and other community organizations that provide services to persons who use drugs can also provide opportunities for STD prevention and case-finding, through promotion of safer sex practices, condom distribution, and testing for syphilis and other sexually transmitted infections.

The findings in this report are subject to at least three limitations. First, syphilis case report data do not include data on opioid use other than heroin, so it was not possible to assess nonheroin opioid use among persons with syphilis. Second, cases with incomplete data on variables of interest were excluded from this analysis. Overall, depending on the year and variable, 18%–25% of reported cases of P&S syphilis among women, MSW, and MSM were missing data on methamphetamine use, sex with a person who injects drugs, injection drug use, or heroin use during 2013–2017. If persons whose records had missing data were less likely to have a risk factor, it is possible that this analysis overestimated the prevalence of these risk factors among persons with syphilis. Finally, because of stigma surrounding these risk behaviors, some persons might have been reluctant to disclose drug use, leading to misclassification and underestimates of the true percentage of persons with syphilis who used these drugs.

The recent increases in heterosexual syphilis, together with the concurrent increases in percentage of persons with P&S syphilis reporting methamphetamine use, sex with a person who injects drugs, injection drug use, and heroin use, are causes for concern. Heterosexual syphilis and drug use, particularly methamphetamine use, are connected and interrelated epidemics in the United States. Collaboration between STD control programs and partners that provide services for persons with substance use disorders will be essential to address recent increases in heterosexual syphilis and link patients to clinical and prevention services.

**Summary**

**What is already known about this topic?**

During 2013–2017, the primary and secondary (P&S) syphilis rate increased 72.7% nationally and 155.6% among women.

**What is added by this report?**

During 2013–2017, reported methamphetamine, injection drug, and heroin use increased substantially among women and heterosexual men with P&S syphilis.

**What are the implications for public health practice?**

Heterosexual syphilis transmission and drug use, particularly methamphetamine use, are intersecting epidemics. Collaboration between sexually transmitted disease control programs and substance use disorder services providers will be essential to address recent increases in heterosexual syphilis transmission. Linking syphilis patients with substance use disorders to behavioral health services and providing syphilis screening for persons receiving substance use disorder services are needed to address these co-occurring conditions.
